# Impact of NSCLC metabolic remodeling on immunotherapy effectiveness

**DOI:** 10.1186/s40364-022-00412-1

**Published:** 2022-08-29

**Authors:** Lulu Lv, Ruo han Huang, Jiale Li, Jing Xu, Wen Gao

**Affiliations:** Department of Oncology, The First Affiliated Hospital of Nanjing Medical University, 300 Guangzhou Road, Nanjing, 210029 China

**Keywords:** Metabolic reprogramming, TME, NSCLC, Immunotherapy

## Abstract

It is known that metabolic reprogramming (MR) contributes to tumorigenesis through the activation of processes that support survival of cells, proliferation, and grow in the tumor microenvironment. In order to keep the tumor proliferating at a high rate, metabolic pathways must be upregulated, and tumor metabolism must be adapted to meet this requirement. Additionally, immune cells engage in metabolic remodeling to maintain body and self-health. With the advent of immunotherapy, the fate of individuals suffering from non-small cell lung cancer (NSCLC) has been transformed dramatically. MR may have a profound influence on their prognosis. The aim of this review is to summarize current research advancements in metabolic reprogramming and their impact on immunotherapy in NSCLC. Moreover, we talk about promising approaches targeting and manipulating metabolic pathways to improve cancer immunotherapy’s effectiveness in NSCLC.

## Introduction

As the main reason for cancer-associated deaths, universally non-small cell lung cancer (NSCLC) has seen a remarkable increase in incidence rate for the past few years [1, 2]. According to histological and pathological classification, lung cancer is mainly divided into lung adenocarcinoma (LUAD), lung squamous cell carcinoma (LUSC), large cell carcinoma and other rare types [3]. With its low 5-year survival rate and high mortality rate, advanced non-small cell lung cancer keeps being a big challenge of oncology [4]. To date, treatment options available for NSCLC incorporate surgery, adjuvant therapy, chemotherapy, radiotherapy, and immunotherapy. Nevertheless, effective therapies of NSCLC, especially for advanced stage cancers, is still lacking [5]. Consequently, to create alternative therapies for such cancer is in urgent need. Compared with traditional treatments, cancer immunotherapy represented by PD-1 blockade has led to a model transition in cancer treatment owing to better survival, less side effects and wider scope of application [6, 7]. So far, two monoclonal antibodies blocking PD-1 (nivolumab, pembrolizumab) together with two blocking PD-L1 (atezolizumab, durvalumab) gain the approval from the FDA to be applied to first-line regimens without prior platinum-containing chemotherapy and for second-line regimens after failure of platinum-containing chemotherapy [8–11].

However, responses to immune checkpoint blockade (ICB) therapy are not widespread, with many patients displaying primary resistance to ICB monotherapy [12]. In some patients, ICB treatment may even result in immune activation against specific organs immunotherapeutic-induced adverse events (irAE). In general, irAEs colocalize with barrier tissues (gut, lungs, and skin) as well as with endocrine tissues (pancreas and thyroid), but importantly, the most common irAE differ from drug to drug [13, 14]. It can be challenging to use combined ICB in clinical settings due to its higher number of side effects. Studies are underway to develop effective approaches to minimize IRAEs without compromising anti-tumor immunity.

With further understanding of immunoregulatory mechanisms in the range of the tumor microenvironment (TME)，the answers to these questions will emerge. A number of elements acting in the TME restrain the curative activities of ICBs. Various findings suggest that immune cells, like Myeloid-derived suppressor cells (MDSC), Tumor-associated macrophages (TAMs), Tumor associated dendritic cells (TADCs) have different potentials in predicting response to anti-PD-(L)1 therapy in patients with NSCLC, respectively. For instance, the immune response to immunotherapy was significantly improved when PMN-MDSC levels were high or the CD8/PMN-MDSC ratio was low [15]. While, TIM-3 expression on lymphocytes and early accumulation of M-MDSC with (Lin*CD33+ CD14 + CD15* HLA-DR*) is associated with resistance to PD-1 blockade [16]. Additionally, the ratio of circulating Treg to G-MDSCs may also affect the response to nivolumab, since patients with a high proportion of circulating Tregs and low proportion of G-MDSCs show improved PFS in patients with NSCLC [17]. In NSCLC, mature DCs are located exclusively within TLSs and are associated with a good prognosis [18]. High DC-LAMP1^+^ mature DCs and High stromal CD561 cells were correlated with prolonged survival in patients with NSCLC [19, 20]. At the same time, High CD1a1^+^LCs together with CD14^+^CD68^low^ interstitial DCs were both associated with longer DSS [21].

Here, we review how the metabolic pattern in lung cancer is remodeled with transformation and malignant progression and how this gives rises to immune escape and resistance to ICBs. We also conclude how these discoveries are applied to strengthen the efficacy of ICBs in patients with NSCLC.

## Glucose metabolism in NSCLC

### Glucose metabolism of tumor cells

**Fig. 1:**
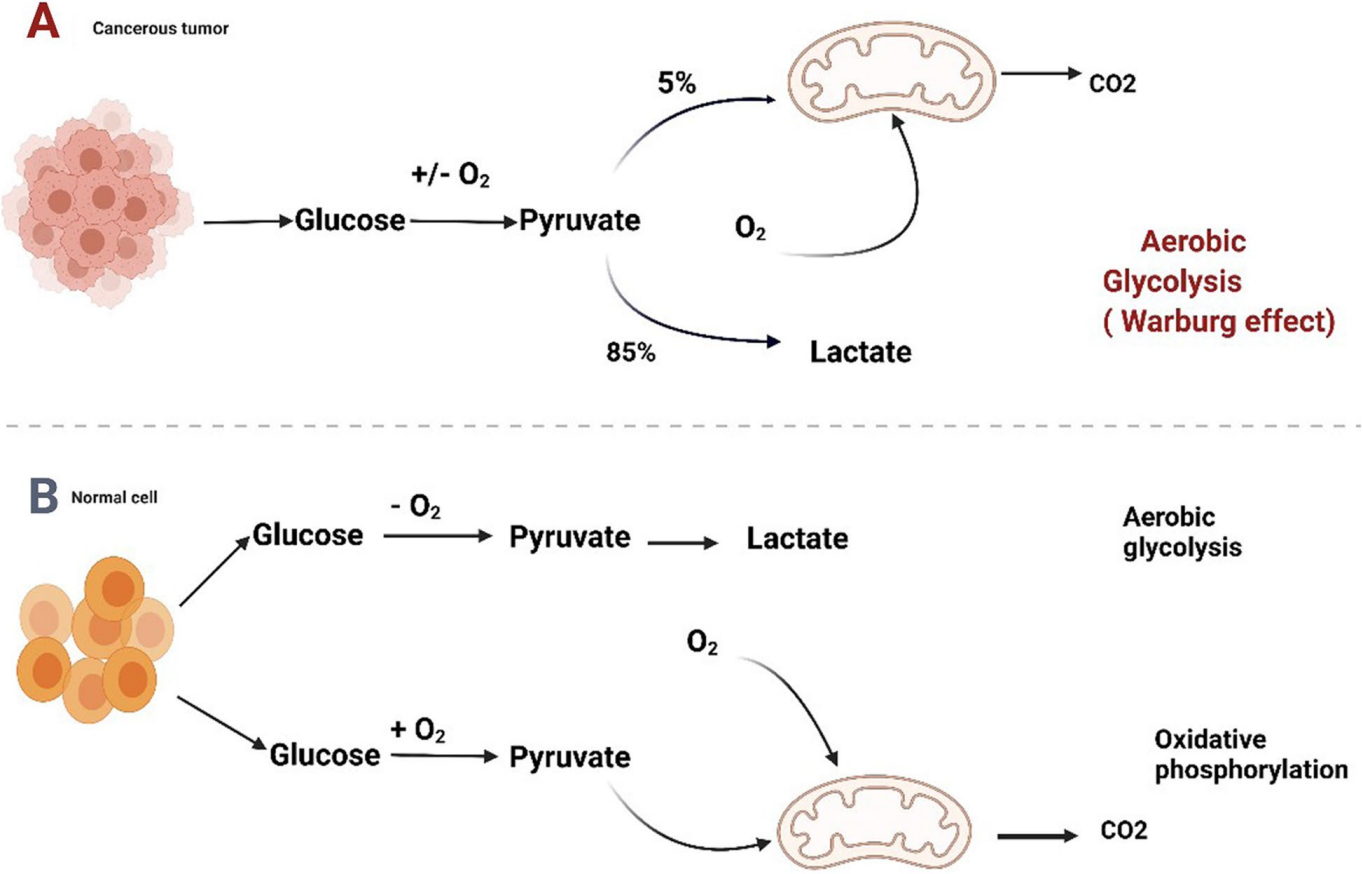
Metabolic control of glucose by cancer cells. Mitochondria are primarily involved in glycosis and the TCA cycle in glucose metabolism. The pathways in tumor cells are generally altered compared to those of normal cells. Figure created using BioRender.com

**Fig. 2:**
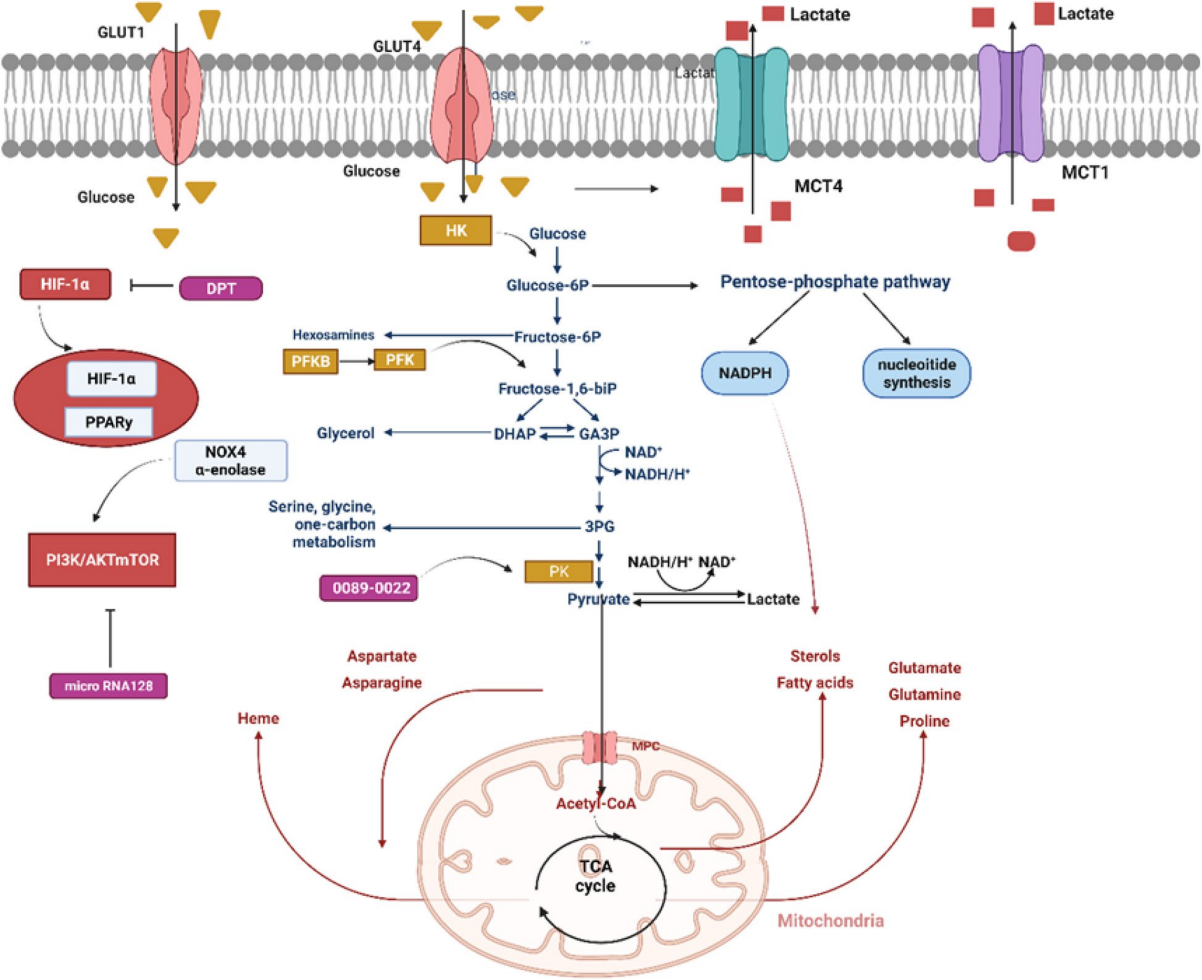
Reprogramming the metabolic process of glucose in lung cancer. In addition to HIF-1α pathway，PPARy and PI3K/AKT/mTOR signaling impair the activity of enzymes and transporters limiting metabolic reprogramming in NSCLC. In the cell, glucose is transported by glucose transporters (GLUT)1 and 4. Glycolysis begins with the phosphorylation of glucose by hexokinase (HK). In the cycle of pentose phosphate (PPP), glucose-6-phosphate is converted into nucleosides and NADPH by glucose-6-phosphate dehydrogenase (G6PD). Dephosphorylating PEP is accomplished by pyruvate kinase (PK) at the end of the glycolytic pathway to synthesize pyruvate and ATP. After that, pyruvate is turned into lactate by-lactate dehydrogenase (LDH). Lactate is delivered to the outside of the cell by monocarboxylate transporters (MCT) 1 and 4. During metabolism, pyruvate can be converted into acetyl coenzyme A (acetyl-CoA), which is following used by the tricarboxylic acid cycle in mitochondria to give rise to ATP and intermediate molecules that are essential to the biosynthesis of both lipids and amino acids. Figure created using BioRender.com

Otto Warburg’s pioneering work displayed in the 1920s that tumor cells consume more glucose than normal cells. The phenomenon went by the name of the aerobic glycolysis or Warburg effect (Fig. 1) [22]. With the physiological condition in oxygenated environment, glucose is metabolized by cells by the course of glycolysis, the tricarboxylic acid (TCA) cycle and oxidative phosphorylation (OXPHOS), and eventually molecular oxidation is completed. This process which is dependent on oxygen is known as oxidative phosphorylation, and 32–38 ATP molecules are ultimately produced from a glucose molecule. However, pyruvate, being converted to lactate, is the byproduct of glycolysis. Merely two molecules of ATP are contained in per mol of glucose. For cancer cells，this metabolic shift includes an upregulation of biosynthetic along with bioenergetic pathways in order to maintain high proliferative rate and adapt metabolism (shown in Fig. 2).

### Effect of glucose metabolism in NSCLC

There is more and more evidence suggesting metabolic remodeling is activated deeply in carcinogenesis and malignant progression in lung cancer (LC) [23]. High glucose uptake is discovered in NSCLC via positron emission tomography/computed tomography (PET/CT). Patients who has NSCLC with high glucose uptake can be identified with glucose-analog fluorodeoxyglucose (18F-FDG) PET/CT scans, which is on the rise as a potential instrument to choose patients for metabolically targeted anti-tumor treatment [24]. fLC tissue illustrates growing glucose contribution to tricarboxylic acid cycle (TCA) cycle in comparison with normal lung tissue, and lung cancer cells embody diverse glycolysis rates and mitochondrial abilities [25]. In addition, in LC cells, pyruvate carboxylase (PC) and pyruvate were overexpressed compared to normal lung tissues. Even when tumor cells are cultured without glucose, activation of alternative pathways for phosphopentose (PPP) still acts a vital part in tumorigenesis [26]. Moreover, PPP activation can trigger a good deal of glutathione and nicotinamide adenine dinucleotide phosphate (NADPH), whose oxidase activity and expression are associated with malignant biological behavior of LC. The function of inhibiting NADPH oxidase downregulates the proliferation and invasion of LC [27, 28]. Thus, Lung cancer cells alter their metabolism by taking away glucose for energy production through glycolysis, generating biomass through PPP and deprotonation, and counteracting oxidative stress through PPP.

### Regulation of glucose transport and metabolism in NSCLC

There are two pathways of cellular glycolysis: oxygen-dependent and oxygen-independent pathway. These two pathways both depend on some familiar glucose transporters and glycolytic enzymes (Fig. 2). The oxygen-dependent mechanisms are mediated by transcription factor hypoxia-inducible factor 1-alpha (HIF-1α) pathway [29]. It has been shown that hypoxia promotes glycogen accumulation in cells through HIF-1α stabilization [30]. The recent study showed DPT (Deoxypodophyllotoxin) serves as an anticancer agent in NSCLC by suppressing HIF-1α activation at the protein level in NSCLC cells to reduce glycolysis [31]. Another study demonstrates that AC020978’s part in advancing cell growth and metabolic reprogramming in NSCLC, which uncovered that AC020978 could regulate PKM2-enhanced HIF-1α transcription activity [32].

Activation of the PI3K-AKT-mTOR signaling pathway mainly mediates oxygen non-dependent mechanisms of glucose utilization in LC [33, 34]. From a previous report,50–73% of NSCLC patients with poor prognosis exhibit high expression of AKT [35], while only minor patients with NSCLC embody mutations of PI3K and AKT.

Aside from activation of mutations, other molecules could also foster PI3K/AKT/MTOR signaling in LC. For instance, microRNA128 (Mir-128) plays an inhibitory role in LC progression by way of inhibition of AKT expression,thereby down-regulating glycolysis [36]. However, some other molecules have been reported activate glycolysis metabolism of LC by targeting PI3K/AKT pathway, such as oxidase 4 (NOX4) and α-enolase [37, 38].

Glucose transporters (GLUTs) belong to a protein family which is beneficial to the transferring of glucose into the blood. Several studies have shown that higher level of GLUT-1 protein was spotted to be remarkably connected with resistance to radiotherapy and poor disease particular overall survival of lung cancer [39, 40]. However, another study performed by Osugi et al. indicated that NSCLC patients with GLUT-1 expression failed to independently display poorer overall survival in comparison to GLUT1-negative patients [41]. Furthermore, GLUT-4 was also identified to be an appropriate potential target for epigenetic treatment or metabolic targeting in the management and NSCLC therapy [42].

Hexokinase (HK) is the first rate-limiting enzyme when cells begin glycolysis. HK is composed of four isoforms featured with diverse functions along with cellular positions. HK-II serves as an enzyme which catalyzes the phosphorylation of glucose, and it is the first step of glycolytic rate [43]. The expression levels of the HK-II protein in aggressive cancer cells greatly exceed those in normal cells [44]. Recently，supramolecular assemblies of new-type amphiphilic cell-penetrating peptides in order to target cancer cell mitochondria stand for an emerging instrument for suppressing tumor growth. The adopted strategy is designed to amplify the apoptotic stimuli by impairing the mitochondrial VDAC1 (voltage-dependent anion channel-1)-hexokinase-II (HK-II) interaction [45].

NSCLC cells have overexpressed phosphofructokinase (PFK) (Fig. 1) as well, driving glycolytic flux to grow. Among the members of PFKFB, PFKFB3 appears higher level of (740-fold) kinase activity and lesser level of bisphosphatase activity. Based on previous studies, PFKFB3 was widely expressed in diverse organs and tumor cells, including lung, gastric, breast, ovarian, and thyroid carcinomas. It causes shifts in metabolism, giving rise to the proliferation and survival of tumor cells [46, 47]. A recent study also demonstrates that targeting PFKFB3 restrained cell viability and glycolytic activity, which may be a promising therapeutic strategy in treating lung adenocarcinoma [48]. Another PFK-1 family member, PFKP, was found to be up-regulated in both NSCLC tissues and cell lines and is correlated with lung cancer cell proliferation and patient prognosis [49].

The M2 isoform of pyruvate kinase (PKM2), as a glycolytic terminal enzyme, catalyzes the last step of glycolysis, shifting the phosphate from phosphoenolpyruvate (PEP) to adenosine diphosphate (ADP) [43]. It has become a vital element that adjusts aerobic glycolysis in cancer cells [50]. The strategies of PKM2 inhibition or silencing [51] as well as activation [52, 53] have shared equal debates in literature owing to potential of treatment in limiting tumor growth.

Gopinath Prakasam found that knockdown of AMPK in cells silenced for PKM2 or PKM1 took on inhibiting growth and contributed to apoptosis [54]. While another study carried out by Li.et al. showed that a potential PKM2 activator, 0089–0022, serves as a promising anti-cancer therapy candidate in NSCLC [55].

Due to the Warburg effect, a large amount of lactic acid is synthesized from pyruvate [22]. Lactate generated by LDH is shifted by monocarboxylate anion transporters (MCT) so that an alkaline internal environment is maintained. This will bring benefits to metabolism [56]. As is well-known to all that MCT4 transfers lactate out of the cell and MCT1 moves the entry of lactate to tumor cells [57]. A previous study had shown cellular expression levels of MCT1 and MCT4 were in relation to invasion activity. This suggests inhibitors of the MCT maybe offer a new-model strategy in order to hinder cancer metastasis [58].

In short, there is evidence demonstrates that LC employs HIF-1α to improve glycolytic flux and to neutralize ROS via the upregulated activity of glucose importers (GLUT1, GLUT4), glycolytic enzymes (PFK, PK), and lactate transporters (MCT1, MCT4). Suppressing metabolic enzymes activity involved in glycolysis and lactate production may become promising new alternative therapeutic targets for lung cancer. Up to date, the majority of prior available evidence was concentrated on small single studies, which only involve partly LC metabolism in a limited number of primary human LC cell lines. Before transforming into clinical trials, more comprehensive preclinical investigations on LC metabolism in divergent phases of the disease are in demanded to enhance the effectiveness of these findings.

## Impact of metabolism reprogramming on cells of the tumor microenvironment and immunotherapy in NSCLC

### Glucose and glycolysis

**Fig. 3:**
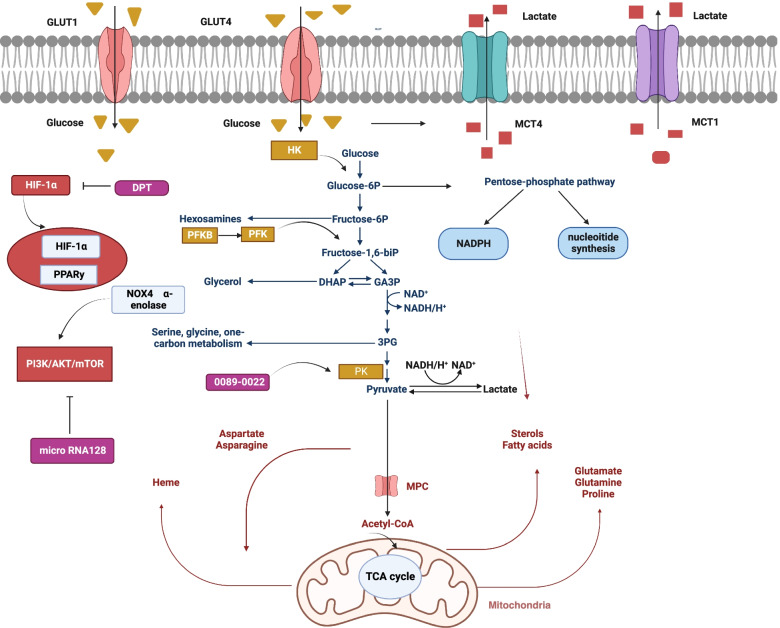
Aspects of glucose metabolism that may serve as therapeutic targets. **A** Cancer patients have successfully used immune-based interventions, including immune checkpoint inhibitors, to treat a variety of cancers. Low glucose, acidity and lactic acid in tumor microenvironment lead to enhanced expression of checkpoint receptors. In turn, this results in reduced glycosis and increased FAO, therefore causing immunosuppression. Immunotherapy targeted at these checkpoint receptors has been successful in restoring glycolysis to immune cells, which promotes anti-tumor immunity against tumors. **B** In the TME, increasing lactate promotes the survival of immune suppressive T cells and restraing the function of T effector. Lactate accumulation can be reduced by inhibiting lactate-producing enzymes, inhibiting lactate transporters or neutralizing acid-induced by lactic acid. These strategies have been proven effective in improving anti-tumor immunity. Figure created using BioRender.com

The tumor microenvironment (TME) plays a vital role in tumor behavior and therapeutic effect [59]. Meanwhile, cancer cells can regulate a well-characterized metabolic phenotype that can deeply affect TME [60]. In addition, an emerging theme is that metabolic phenotypes are tissue specific and cancer subtype specific. As a highly heterogeneous disease covering a heterogeneous population, NSCLC is blessed with a complicated system to identify the disease state and progression [61]. Tumor-induced TME metabolic reprogramming as shown in Fig. 3 appears to be more common.

With the increase of metabolic activity of tumor cells, glucose and amino acids in tumor microenvironment were significantly deficient. Glucose utilization by tumors metabolically limits T cells, which paralyzes their ability to maintain aerobic glycolysis and anti-tumor activity [62–64]. This is partly attributed to T cells, like tumor cells, being metabolically dependent on glycolysis. Further, T cell metabolism is regulated by HIF-1α, PPARy signaling and the transcription factor C-MYC-associated pathway and nuclear receptor family pathway [65, 66].

**Fig. 4:**
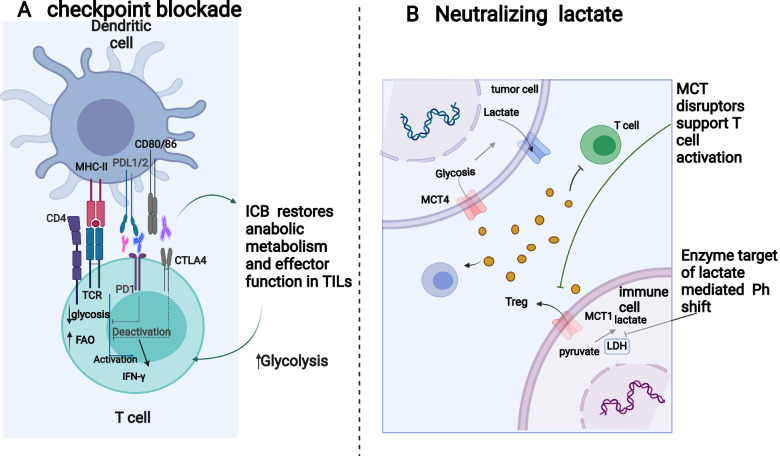
The effects of lung cancer metabolism on immune cells within the tumor microenvironment (TME). Cancerous TME develop in lungs, lacking glucose and tryptophan, while immune-suppressive molecules such as lactate and kynurenine accumulate. In the tumor microenvironment, the high levels of lactate inhibit the tumor immune responses by (1) polarizing macrophages toward the suppressive M2 phenotypes, as well as (2) preventing monocyte migration and dendritic cell differentiation. (3) The presence of excessive lactate also inhibits the function of CD8+ T cell directly. Besides kynurenine, tumors are also known to repress T-cell activity, as is tumor-induced tryptophan deficiency and glucose deprivation. Figure created using BioRender.com

However, tumor-restricted glucose utilization would not totally make T cells disrupted, presumably because T cells seek for available metabolic sources. For example，mitochondrial activation of CD8 + T cells by PPAR-γ agonists strengthens the anti-tumor immunity in T cells during PD-1 blockade [66]. Another experiment has demonstrated that reactivation of depleted T cells relies on reserve of lipids by fatty acid oxidation in T lymphocytes receiving PD-1 signals [67]. Thus, blocking these pathways with checkpoint inhibitors can partially rescue glycolysis and biosynthesis, thereby reversing the effector function of CD8 + T cells (Fig. 4).

On the basis of the previous studies, declining metabolic burden on effector T cells, that lies in tumor microenvironment, might cause durable and steady anti-tumor immune responses. For instance, inhibition of GLUT1 receptors may contribute to more useful T cells and strengthen anti-tumor immune responses, or under the circumstance of glycolysis inhibitors like 2-deoxyglucose (2-DG), antitumor function can be enhanced in primed T cells [68, 69].However，several other studies have demonstrated that inhibiting nutrient transporters and enzymes got involved in glucose metabolism of CD8 + T cells could regulate T cell differentiation and inhibit CD8 + T cell function under low-glucose conditions [70].

The Treg cells accumulate in the TME and play a critical role in dampening antitumor effect. According to growing evidence, Tregs are able to differentitate and survive due to low glucose availability imposed by tumor condition. This extreme environment requires Tregs to utilize oxidative phosphorylation (OXPHOS) as a source of energy. In fact, lactate and kynurenin, metabolic waste products of glycolysis pathways, inhibit conventional T cell activation and cytotoxicity in Treg cells [71–73]. Reprogramming tumor cells, for example, inhibits the infiltration of effector T cells (Teffs) or induces apoptosis, enhances the differentiation of regulatory T cell (Tregs). As a result of reprogramming tumor cells, lactic acid accumulates and carbon dioxide is released, thereby suppressing the immune system [74].

Contrary to CD8+ and CD4+ T cells, Treg metabolism dependes on external factors which include nutrient availability as well as TCR triggering and cytokine milieu, [75, 76]. It is likely that Treg cells are not affected by glucose competition in the tumor site because they are able to utilize alternative to glucose for energy [77]. Rather than glycolysis, the metabolism of Tregs is primarily based on the oxidation of fatty acids [78, 79]. Tregs rely primarily on FAO for self-maintenance, and they exhibit low mTOR activity, so these fatty acids provide the perfect soil for Treg maintenance [80].

It has been demonstrated that fatty acids, combined with in vitro inhibition of glucose uptake and glucose oxidation, lead to Treg differentiation [81]. Moreover, lipid uptake and oxidation are mandatory for the expression of Foxp3, as demonstrated in murine models [81]. In hypoxic tumors, hypoxia-inducible factor (HIF)-1* elicits pyruvate to exit mitochondria with OXPHOS, causing Tregs reliant upon fatty acids for mitochondrial metabolism. As a result, FAO plays an important role in cancer metabolism of Tregs [82].

### Effects of acidic extracellular microenvironment on immune cells

One main driving force of the metabolic remodeling appearing in the TME is without doubt rendered by hypoxia and accumulation of lactate. Lactate production is able to be higher level (40-fold) in tumor cells, and lactate dehydrogenase (LDH) display a positive relationship with tumor volume and clinical severity, as well as prognosis [83, 84].

Table 1 summarizes the effects of tumor-derived lactic acid on tumor-infiltrating lymphocytes in TME.

**Table 1:** The impacts of lactate and acidification on immune cells in the TME

Immune cells	Effects
TADCs	Inhibition monocyte activation [64]
	Inhibition antigen presentation [65]
MDSCs	Immunosuppressive microenvironment [66]
	Cancer aggressiveness [66]
NK cells	Inhibition of effector functions [66]
TAMs	Induction of M2 polarization [72, 73]
Treg cells	Increase in Treg proliferation [76]
	Enhancement of immunosuppressive effect [76]
CD8 + T cells	Inhibition of effector functions [74]

As antigen presentation cells, tumor associated dendritic cells (TADCs) initiate and enhance antitumor immune responses. TADCs promote antitumor immune surveillance, as they are able to exhibit neoantigens to T cells, thereby initiating a T-cell-mediated immune response [85]. Studies have shown that lactic acid accumulation has a direct immunosuppressive effect on immune cells. Lactic acid inhibits monocyte activation and dendritic cell antigen presentation [85, 86].

Myeloid-derived suppressor cells (MDSC) are key components of protumor immune responses and their accumulation in immune organs relate to immunosuppression and cancer aggressiveness. Tumoral lactate production was also found to increases MDSCs and suppresses natural killer (NK) cell function, further accelerating the immunosuppressive microenvironment [87]. A recent study conducted by Baumann found that MDSC blocks T cells by transferring the glycolytic metabolite methylglyoxal, which acts as an immunosuppressant by consuming L-arginine in CD8+ T cells [88].

Tumor-associated macrophages (TAMs) serve as main components in TME [89].TAM, being highly plastic, is able to polarize to two primary phenotypes: the antitumor M1 (TAM1) together with the protumor M2 (TAM2). Several experiments and clinical trials demonstrate that TAMs are primarily of the M2 phenotype, which drive tumor progression and metastasis [90, 91] as well as suppression of antitumor immune responses [92].

Tumor-cell-derived lactic acid facilitate M2-polarization of TAMs through improved arginase and HIF-1α stabilization [93, 94]. After we treated TAMs from patients either lactate or conditioned medium from two tumor cell lines: the Lewis lung carcinoma (LLC) and the melanoma cell lines, TAMs show enhanced expression of HIF-1α and M2-polarization [93].

Therefore, lactic acid produced by cancer cells has a direct immunosuppressive effect, further initiating MDSC-mediated immunosuppression and driving M2 polarization by inhibiting the differentiation of monocyte derived dendritic cells. Tumor-derived lactate can also decrease T cell function by reducing lactate export through MCT1, which inhibits their capability to retain aerobic glycolysis [95]. Additionally, inhibition of effector T cell and boost of Treg in TME by lactate lead to enhanced immunosuppressive microenvironment [96, 97]. Hence, neutralization of an acidic environment is probably to have a significant implication on further the efficacy of ICB (Fig. 3).

Meanwhile, in a recent preclinical study, buffering the TME with bicarbonate administration able to limit tumor growth and improve antitumor responses in animals when merged with either anti-PD-1antibodies (Abs), anti-CTLA or adoptive T cell transfer in a melanoma model [98] .Although molecular mechanism underlying this combination were not clear，these results imply that reversing the acidic TME may increase the selectivity of treatments and meanwhile improve ICB therapy by increasing tumor infiltration by activated T lymphocytes. Fig. 4.

#### Amino acids metabolism

NSCLC has metabolically increased dependence on glucose and cystine or glutamine. Some amino acids have been studied as anti-cancer targets for drug discovery, involving glutamine and L-tryptophan.

Smoking induces the expression of cystine-glutamate antiporter xCT (SLC7A11) in NSCLC cells. Hence, one approach is to deprive cancer cells of up taking cystine by regulating glutaminolysis. Sulfasalazine (SASP), a FDA-approved medication owns suppressive influence on the function of xCT, which inhibits cystine uptake and contribute to cystine depletion [99, 100]. By reinforcing the correlation of xCT, the combination of tumor cell xCT deficiency with anti-CTLA, accelerated the frequencies and anti-tumor activity and persistence [101, 102].

An available strategy is to induce cancer cells glutamine deprivation by reducing glutamine uptake. CB-839 (Telaglenestat) is one of the most effective and selective GSL1(glutaminase1) inhibitors. It has been shown to suppress growth in non-small-lung-cancer, synergize with immune checkpoint blockade Nivolumab or pembrolizumab [103].

Indoleamine-2,3-dioxygenase 1 (IDO1) catalyzes the conversion of tryptophan into kynurenine which lead to an immune-suppressive TME and thus contributing to tumor growth [104]. Growing IDO1 activity prevents T effector activation,dampens NK cell function, facilitates Treg activation, and drives the expansion and activation of DCs and MDSCs [105, 106]. Accordingly, IDO is an appealing therapeutic target for anti-tumor drug discovery. So, can we potentiate the efficacy of ICB for NSCLC patients by restoring TME tryptophan levels and inhibiting IDO1-dependent tryptophan metabolism? Several clinical trials have been investigating it and more comprehensive investigations are in needed. Epacadostat (Keynote-037) is one of the clinical-stage IDO1 inhibitors (Table 1). Its combination with immune checkpoint inhibitor pembrolizumab yielded promising data in solid tumors (including NSCLC). However, in the phase III trial (KEYNOTE-252), Epacadostat failed to meet their endpoint in unresectable or metastatic melanoma [107, 108].

Meanwhile, several clinical trials are under way to test the combination of IDO1 inhibitor with antitumor vaccines. A phase I IDO-derived peptide vaccination study was performed in patients with stage III/IV NSCLC based on long-term follow up (NCT01219348). Here, we present the long-term clinical and immunological outcomes of the vaccine-based strategy [109, 110]. All these has prompted researchers to make breakthrough about IDO1 inhibition and inhibitory metabolites tryptophan, thereby identifying patients candidates most susceptible to benefit from cancer immunotherapy.

Finally, Metformin, the old anti-diabetic drug, has also found its new place when combining with immunotherapy. According to studies in vivo and in vitro, targeting AMPK could be an meaningful strategy to augment immune checkpoint blockade efficiency. A phase II trial is evaluating the initial efficacy of metformin combined with nivolumab to treat metastatic lung cancer patients (NCT03048500).

Considering the metabolic interplay between cancer and immunes cells, the immune checkpoint blockade can synergize with metabolic intervention therapies. Table 2 lists the tentative therapeutic targets that integrate metabolism with immunotherapy and will be further discussed below. Table 3 lists novel metabolic reprogramming immunotherapies that are in the preclinic.

**Table 2:** Metabolic reprogramming in immunotherapy clinical trials

Medications	Target	Combination Therapy	Context	identifier/reference
Metformin	AMPK	Nivolumab (anti-PD1)	NSCLC	NCT03048500
epacadostat	IDO	Pembrolizumab	NSCLC	Keynote-037
epacadostat	IDO	Pembrolizumab + Platinum doublet	NSCLC	NCT03322566
epacadostat	IDO	Pembrolizumab	NSCLC	NCT03322540
IO102	IDO	Pembrolizumab ± Platinum doublet	NSCLC	NCT T03562871
ID05-peptide IDO	IDO	vaccine-based	NSCLC	NCT01219348
Telaglenestat (CB-839)	Glutaminase1 inhibitor	Nivolumab	NSCLC	NCT02771626
(CB-839)	Glutaminase	pembrolizumab	NSCLC	NCT04265534

**Table 3:** Metabolic reprogramming immunotherapies in the preclinic

**Inhibitors**	**Targets**	**Cancer types**	**References**
Dichloroacetic acid	PDH	breast cancer	[111]
Dimethyl fumarate	G6PD	breast cancer	[112]
L-Arg bacteria	L-arginine	Colon Cancer	[113]
oxamate	LDH	lung cancer cells	[114]
CB-839	Glutaminase	Melanoma mouse	[115]
Protein phosphatase 2A	PPP	SCLC	[116]
AZD3965	MCT1	TC-1 cancer cells	[117]

## Conclusion

TME consists of a variety of cell populations and matrices, in which the bioenergetic requirements of quickly dividing cancer cells and immune cells are pitted against one another for essential nutrients. The metabolic reprogramming of lung cells is magnified by hypoxia, PPARy and PI3K/mTOR/AKT signaling-mediated overexpression of PPP, as well as glycolysis and lactate production as well.

Several immunotherapeutic options have been explored in recent decades in order to block or reprogram TAMs’ or MDSCs’ immunosuppressive activities. Many therapeutic strategies have utilized TAMs and M-MDSCs or their functional mediators as direct targets, including inhibiting CSF1 (M-CSF) interactions with its receptor CSF1R,by small molecules or neutralizing anti-CSF1R (e.g., PLX3397, GW2580, IMC-CS4, AMG820) or anti-CSF1 (e.g., emactuzumab, cabiralizumab) monoclonal antibodies (mAbs), by blocking the production of anti-CSF1R or anti-CSF1 monoclonal antibodies, inhibiting the M2-like phenotypes of TAMs, increasing infiltrating CD8+ T cells, and improving the immune response to ICB. The further investigation of synergistic effects of checkpoint blockade-based immunotherapies on TAMs or MDSCs will improve ongoing immunotherapeutics.

In addition to TAMs and MDSCs, DCs,as the most powerful antigen-presenting cells (APCs) of the immune system, also suffer from metabolic disorders. When DCs transition from an immature state to a mature state, they demonstrate metabolic plasticity. FAO drives ATP production in immature DCs via OXPHOS. Tumor-associated DCs accumulate lipids, which are negative regulators of their ability to process antigens via MHC class II and to stimulate allogenic T cells. There is now a need for new studies to evaluate how anticancer therapies affect myelopoiesis and immunomodulation, as well as their interactions with metabolism in the host.

Energy and nutrients deprivation in the TME synergy with immune-suppressive molecules like lactic acid jointly promoting the suppressive TME. This further exacerbate immune escape. Strategies for combing ICB with LC metabolism meet the metabolic requirements of immune cells like M1 macrophages or CD8+ T cells, boosting anticancer immunity. Recently，clinical studies in relation to the integration of ICB with drugs tumor-targeting are under way. IDO-inhibition could overcome the detrimental effects of tryptophan depletion and kynurenine accumulation and balance tryptophan-kynurenine pathway.

Simultaneous inhibition of glycolysis and lactic acid production in LC cells seems to be an effective method to improve the efficacy of immunotherapy. But so far, tumor targeting and localization targeting to protect non-cancer cells from glucose starvation still need to be further explored.

In a word, disrupting LC metabolism perhaps be a promising option to promote cancer immunotherapies. Novel therapeutic targets are under development in preclinical models, at the same time, retooling existing drugs for optimized ICB should be investigated as well.

## Data Availability

Not applicable.

## Abbreviations

MR: Metabolic reprogramming
NSCLC: Non-small cell lung cancer
LUAD: Lung adenocarcinoma
LUSC: Lung squamous cell carcinoma
ICB: Immune checkpoint blockade
irAE: Immunotherapeutic-induced adverse events
TME: Tumor microenvironment
TCA: Tricarboxylic acid
OXPHOS: Oxidative phosphorylation
LC: Lung cancer
PET/CT: Positron emission tomography/computed tomography
18F-FDG: Glucose-analog fluorodeoxyglucose
PC: Pyruvate carboxylase
PPP: Phosphopentose
NADPH: nicotinamide adenine dinucleotide phosphate
HIF-1α: transcription factor hypoxia-inducible factor 1-alpha
DPT: Deoxypodophyllotoxin
Mir-128: microRNA128
NOX4: Oxidase 4
GLUTS: Glucose transporters
HK: Hexokinase
VDAC1: Voltage-dependent anion channel-1
PEK: Phosphofructokinase
PKM2: M2 isoform of pyruvate kinase
PEP: Phosphoenolpyruvate
ADP: Adenosine diphosphate
MCT: Monocarboxylate anion transporters
2-DG: 2-deoxyglucose
LDH: Lactate dehydrogenase
TADCs: Tumor associated dendritic cells
MDSC: Myeloid-derived suppressor cells
NK: natural killer
TAMs: Tumor-associated macrophages
Teffs: Effector T cells (Teffs)
LLC: The Lewis lung carcinoma
SAPS: Sulfasalazine
GSL1: Glutaminase1
IDO1: Indoleamine-2,3-dioxygenase 1
